# Physical and mental health of 40,000 older women in England during the COVID-19 pandemic (2020–2021)

**DOI:** 10.1371/journal.pone.0307106

**Published:** 2024-07-18

**Authors:** Sarah Floud, Carol Hermon, Gillian K. Reeves

**Affiliations:** Nuffield Department of Population Health, Cancer Epidemiology Unit, University of Oxford, Oxford, United Kingdom; The University of York, UNITED KINGDOM OF GREAT BRITAIN AND NORTHERN IRELAND

## Abstract

**Background:**

To assess factors associated with perceived changes in physical and mental health and with delays in seeking healthcare during the second and third COVID-19 lockdowns in England (2020–2021).

**Methods:**

An online survey of Million Women Study participants collected data on 44,523 women, mean age 76 (SD = 4), October 2020—May 2021. These data were linked to data collected prospectively on Million Women Study participants at recruitment in median year 1998 and at re-surveys in 2011–2013, as well as to hospital admission data from 2017–2019.

**Results:**

Of 40,821 participants with complete data on the outcomes of interest, 28% reported worse physical health and 26% worse mental health. After adjustment for age, region, education and survey period, poor/fair self-rated health (adjusted OR 2.71, 95% CI 2.52–2.91), having been told to shield (1.92, 1.79–2.05), obesity (2.17, 2.04–2.31) and other measures of poor health prior to the outbreak were all strongly related to worse physical health, as was being an informal carer (1.47, 1.38–1.56) and having a COVID-19 infection (1.64, 1.53–1.77). Depression (2.31, 2.06–2.58), poor/fair self-rated health (1.98, 1.84–2.13) and being an informal carer (1.69, 95% CI 1.58–1.80) were the factors most strongly related to worse mental health. Having poor/fair self-rated health (2.22, 2.05–2.40), obesity (1.58, 1.47–1.70) and being an informal carer (1.45, 1.34–1.56) were all strongly related to delaying seeking medical care. These associations remained essentially unchanged after exclusion of participants who had a COVID-19 infection.

**Conclusions:**

In a large sample of older women in England, just over a quarter reported a deterioration in their physical and mental health during the national lockdowns. In addition to the expected effect of a COVID-19 infection on physical health, the groups who were most likely to report such a deterioration were those with pre-existing morbidity and those who were caring for others as informal carers.

## Introduction

On 30 January 2020, the World Health Organisation (WHO) Director-General declared the COVID-19 outbreak a Public Health Emergency of International Concern, which is the WHO’s highest level of alarm. It soon emerged that older people were at increased risk of severe illness, hospitalisation and death from COVID-19 [1,2]. On 23 March 2020, England went into a national lockdown with most people required to stay at home, and public health guidance classed all people aged over 70, regardless of medical conditions, as clinically vulnerable and advised them to take particular care to minimise contact with others outside their household [3]. In addition, people with serious underlying medical conditions were advised to ’shield’ and not leave their homes for any reason. From 10^th^ May 2020 there was a gradual relaxation of the lockdown rules, which continued until mid-September 2020, when new restrictions were imposed. A second national lockdown occurred from 5^th^ November 2020 to 2^nd^ December 2020 and a third national lockdown was imposed on 6^th^ January 2021, with restrictions eased from 8^th^ March 2021 onwards.

There is evidence that there was a deterioration in mental health in the UK during the first lockdown [4–7] and during the second and third lockdowns [8–12]. Groups that have been identified as being at greater risk of poor mental health during the COVID-19 outbreak include women [11,13–16], younger people [6,13,16], individuals with poorer socio-economic position (SEP) [7], those who were shielding [11,15], those who were living alone [11,13,16], those who had previous mental illness [11] and informal carers [17,18]. Much less is known about which groups were suffering poorer physical health during this period, although there is some evidence that poorer SEP and the provision of informal care were associated with poorer physical health or self-rated health [7,19], and one study has reported greater cognitive decline in those with pre-existing cognitive impairment [20]. In addition, during 2020 there was a drop in the numbers of people contacting their GP and in diagnoses for common and serious conditions [21–23]. Factors that have been reported to be associated with healthcare avoidance in the UK during the pandemic include being female, overweight or in poor health [24].

The aim of this analysis was to describe the impact on older women of the COVID-19 outbreak and the second and third national lockdowns in England, because older women are likely to be over-represented in the vulnerable groups already identified. We used data from the Million Women Study, a population-based cohort study [25]. Between 14 October 2020 and 18 May 2021, 66,516 Million Women Study participants were invited to complete an online questionnaire on their experiences during the COVID-19 pandemic.

This report focuses on perceived changes in physical and mental health and whether socio-demographic, lifestyle and health factors were associated with any perceived changes. A further aim was to identify whether any factors were associated with a delay in seeking healthcare. Importantly, information on most of the factors examined was collected prospectively in the Million Women Study cohort, prior to the COVID-19 pandemic, thereby limiting reporting bias that can affect cross-sectional studies.

## Methods

### Participants

Participants were from the Million Women Study, a population-based prospective cohort study of 1.3 million women in the UK, who were recruited through the National Health Service Breast Cancer Screening Programme in median year 1998 (IQR 1998–1999; range 1996–2001) [25]. In the present investigation, we linked data collected as part of the Million Women Study with participant responses to an online Million Women Study survey completed during the COVID-19 outbreak in the UK. Participants provided written consent for follow-up through their medical records and ethical approval was given by the East of England-Cambridge South Research Ethics Committee (97/5/001). Information on data access is available on the Million Women Study website www.millionwomenstudy.org.

Between 14 October 2020 and 18 May 2021, participants from the Million Women Study, who had previously provided an email address, were invited by email to complete the Million Women Study Coronavirus (COVID-19) online questionnaire. The questionnaire was designed to assess participants’ experiences of the COVID-19 pandemic, and was developed with input from the Million Women Study Participant Panel. It consisted of 19 questions and took approximately 10 minutes to complete.

### Measures of health and help-seeking behaviour

Perceived worsening of physical health, perceived worsening of mental health, perceived improvement in physical health and perceived improvement in mental health were assessed using responses to the following question: “Overall, how would you say the Coronavirus outbreak has affected your life?” Participants were then given different domains to assess, including “Your physical health” and “Your mental health”, with answer options given as follows: “For the worse”, “For the better”, “Stayed the same”. Delay in seeking healthcare was assessed using responses (yes/no) to the following question: "Since the Coronavirus outbreak began, have you delayed going to the doctor for something that you eventually needed treatment for?"

### Factors affecting changes in health and help-seeking behaviour

We assessed factors that might affect changes in health and in help-seeking behaviour, grouping them into three categories: socio-demographic, lifestyle and prior health. Socio-demographic factors included age at completion of the COVID-19 survey (<75, > = 75 years) and educational qualifications (tertiary qualifications, other) which were reported at recruitment in median year 1998. The other socio-demographic factors, living alone and informal caring, were reported by the participants in response to the COVID-19 questionnaire. Participants were asked to state the number of people living together in their household currently, and were categorised as ’living alone’ or ’living with others’. For carer status, participants were deemed to be informal carers if they responded yes to the question: "Before the outbreak, did you regularly care for family members or others because of either long-term physical or mental ill-health/disability or problems related to old age?"

Lifestyle factors were recorded prospectively in 2011–2013 on the Million Women Study resurveys, and included smoking status (never, past, current), body mass index (<25, 25–29, > = 30 kg/m^2^) and alcohol intake (0, 1–7, >7 drinks per week). Self-reports from the 2013 resurvey were prioritised over self-reports from the 2011 resurvey, which were only used when 2013 responses were missing.

Factors representing prior health status were recorded prospectively and included self-rated health (excellent/good, fair/poor) and disability status (receiving disability benefits: yes, no) which were reported in 2011–2013. Admission to hospital in 2017–2019 was used as an indicator of prior health status, using linked Hospital Episode Statistics. Cause-specific admissions during 2017–2019 for ischaemic heart disease (IHD) (I20-I25), hypertension (I10), cancer (C00-C97), asthma (J45) and depression or anxiety (F31-F33, F40, F41) were also investigated separately. Participants were asked on the COVID-19 questionnaire whether they had received a letter or text message asking them to ‘shield’, and this was used as an additional indicator of poor health status, since only those considered to be at high clinical risk of COVID-19 were asked to ‘shield’.

Participants were also asked if they had had a COVID-19 infection that was confirmed by a test or suspected by themselves or their doctor.

### Statistical analysis

We compared the characteristics of participants who responded to the Covid-19 questionnaire with those who did not respond and tested differences using Pearson’s chi-square test. We calculated the overall proportion of women who reported (i) worse physical health, (ii) worse mental health, (iii) improved physical health, (iv) improved mental health and (v) delays in seeking healthcare due to the COVID-19 outbreak and associated lockdowns. We then used logistic regression to assess associations between socio-demographic factors (age, education, living alone, informal carer), lifestyle factors (smoking, BMI, alcohol intake) and prior health status (shielding, self-rated health, receiving disability benefit, hospital admissions, COVID-19 infection) and the odds of changes in physical and mental health and delays in seeking healthcare. In all models adjustment was made for age, education, region at recruitment (London and Southeast, South West, Midlands, North), and survey period (14 October 2020–5 January 2021, 6 January– 7 March 2021, 8 March– 18 May 2021). We also conducted the following sensitivity analyses. We excluded women who reported a definite or probable COVID-19 infection, to exclude the possibility that the results were dominated by COVID-19 infection. Given that participants’ experiences and perceptions of health may have differed over the time period of the study, we tested for heterogeneity in the associations by survey period, and, due to the large number of tests, we adjusted for false discovery rate (FDR) using the Benjamini–Hochberg method, with an FDR threshold of 0.05 set for significance. STATA 18 was used for all analyses.

## Results

A total of 66,516 participants of the Million Women Study were invited to complete the COVID-19 questionnaire and 44,523 women completed it, giving a response rate of 67%. A third (33%) of the participants were invited in late 2020 (14 October 2020–5 January 2021) covering the period of the second national lockdown, 59% were invited during the third national lockdown (6 January– 7 March 2021) and the rest (8%) were invited when the lockdown restrictions were being lifted slowly (8 March– 18 May 2021). Although there were not large differences, the participants who did not respond were more likely to be obese and in poorer health (S1 Table).

Women were excluded from the analysis if they lived in Scotland, since Scotland had different lockdown restrictions (N = 2764), if they did not answer the questions on mental health (N = 593), physical health (N = 110), and delaying seeking healthcare (N = 235). See also the flow chart in the supplementary material (S1 Fig). This left a total of 40,821 women eligible for the analysis and their characteristics are shown in Table 1. The mean age of the participants was 76 (SD = 4); 43% of the respondents had tertiary qualifications, 32% of participants were living alone, and 12% were informal carers. Prior to 2020, over a third (35%) of participants had reported drinking >7 alcohol drinks per week, 16% had BMI > = 30 kg/m^2^ and only 3% were current smokers; and 9% self-reported having poor or fair health, 4% reported receiving disability benefits and 45% had been admitted to hospital during 2017–2019. In addition, 10% of participants reported that they had been told to ’shield’ at the start of the COVID-19 pandemic and 9% reported having had a COVID-19 infection.

**Table 1:** Participant characteristics (40,821 women).

Characteristics	All women
**Socio-demographic factors**	
Age, mean (SD)	76 (SD 4)
<75 yrs, % (n)	49 (19829)
≥75 yrs, %(n)	51 (20992)
Tertiary educational qualifications, % (n)	43 (17476)
Living alone in 2020–2021, % (n)	32 (12911)
Informal carer in early 2020, % (n)	12 (4932)
**Lifestyle factors**	
Current smoker in 2011–13, % (n)	3 (1223)
BMI ≥30 kg/m^2^ in 2011–13, % (n)	16 (6213)
Alcohol intake >7 drinks/week in 2011–13, % (n)	35 (13102)
**Prior health status**	
Poor/fair self-rated health in 2011–13, % (n)	9 (3618)
Receiving disability benefit in 2011–13, % (n)	4 (1614)
Any hospital admission (2017–2019), % (n)	45 (18457)
Hospital admission (2017–2019) with mention of:	
Ischaemic heart disease, % (n)	4 (1546)
Hypertension, % (n)	17 (7027)
Cancer, % (n)	5 (2221)
Asthma, % (n)	5 (1861)
Depression/anxiety, % (n)	3 (1303)
Being told to shield in 2020, % (n)	10 (3965)
Definite/probable COVID-19 infection, % (n)	9 (3636)
**Additional adjustment variables**	
Region at recruitment in 1998:	
London and Southeast, % (n)	32 (13260)
South West, % (n)	24 (9900)
Midlands, % (n)	17 (6956)
North, % (n)	26 (10705)
Survey period	
14 October 2020–5 January 2021	38 (15359)
6 January—7 March 2021	58 (23540)
8 March—18 May 2021	5 (1922)

When asked about changes in their health during the COVID-19 outbreak, 28% of participants reported a worsening of their physical health and 26% reported a worsening of their mental health (Table 2). A relatively small proportion reported these health measures had improved (6% for physical health and 1% for mental health).

**Table 2:** Proportion of participants reporting change in health during the COVID-19 pandemic.

Health	For worse, % (n)	Stayed same, % (n)	For better, % (n)
Physical health	28 (11540)	66 (26757)	6 (2524)
Mental health	26 (10501)	73 (29828)	1 (492)

**Fig 1:**
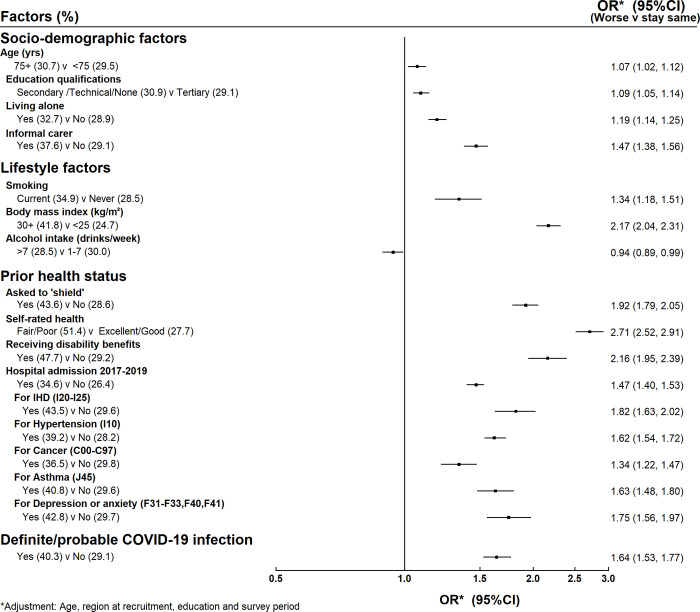
Factors associated with worsening physical health.

**Fig 2:**
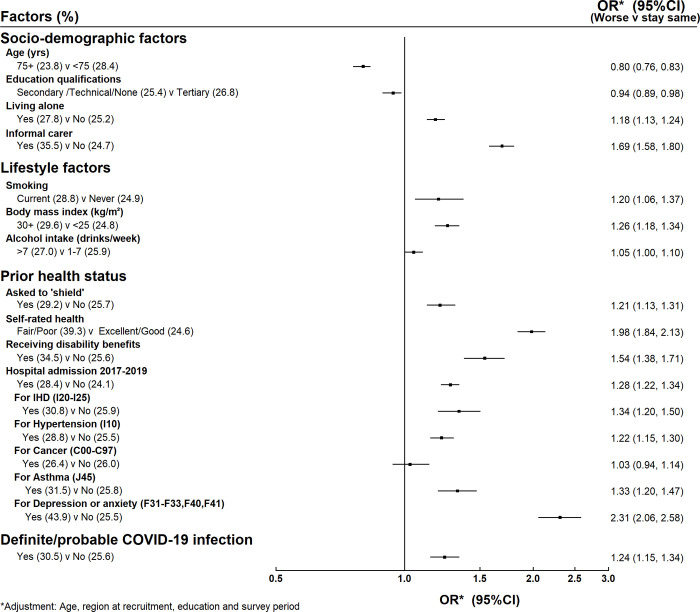
Factors associated with worsening mental health.

Figs 1 and 2 show the associations between the factors of interest (socio-demographic, lifestyle and prior health) and reported worsening of physical health and mental health respectively. Those who were informal carers were much more likely to report worsening physical health than those who were not informal carers (38% compared to 29%, adjusted OR 1.47, 95% CI 1.38–1.56, Fig 1) and they were also more likely to report worsening mental health (36% compared to 25%, adjusted OR 1.69, 95% CI 1.58–1.80, Fig 2). The associations were weaker for the other socio-demographic factors examined.

The main lifestyle factor that was associated with worse physical health was body mass index (BMI). Reported physical health declined with increasing BMI, with women who had a BMI > = 30 kg/m^2^ much more likely to report that their physical health had deteriorated than women with BMI <25 kg/m^2^ (42% compared to 25%, adjusted OR 2.17, 95% CI 2.04–2.31, Fig 1). The lifestyle factor associations with worse mental health were weaker (Fig 2).

Those in poor physical health prior to the pandemic were much more likely to report a worsening of their physical health during the outbreak (Fig 1). For example, those who had been asked to shield were twice as likely to report worsening of their physical health than those who had not been asked to shield (44% vs 29%, adjusted OR 1.92, 95% CI 1.79–2.05), as were those who were disabled prior to the outbreak (48% vs 29%, adjusted OR 2.16, 95% CI 1.95–2.39). Those who rated their health as being fair/poor prior to the outbreak were nearly three times as likely to report worse physical health than those who rated their health as good/excellent (51% vs 28%, adjusted OR 2.71, 95% CI 2.52–2.91).

In the case of worsening mental health, those who had been admitted to hospital during 2017–2019 with depression and anxiety mentioned on their record were much more likely to report worse mental health during the outbreak (44% vs 26%, adjusted OR 2.31, 95% CI 2.06–2.58, Fig 2). Women who had other indicators of poor health status were also more likely to report worse mental health, albeit with not such strong associations as found for worsening physical health, except for those who had been admitted for cancer where there was no association with worsening mental health.

Having had a COVID-19 infection was associated with reporting worse physical health (40% vs 29%, adjusted OR 1.64, 95% CI 1.53–1.77). There was also an association with worse mental health but this was weaker (31% vs 26%, adjusted OR 1.24, 95% CI 1.15–1.34).

Reporting improvements in physical health was rare (6%) and did not differ by many factors, except for a tendency for younger women (aged 68–75) and those with higher education to report improvements in physical health (S2 Fig). Reporting improvements in mental health was very rare (1%) and although there appeared to be some associations, we cannot attach much importance to these given the relatively small numbers (S3 Fig).

**Fig 3:**
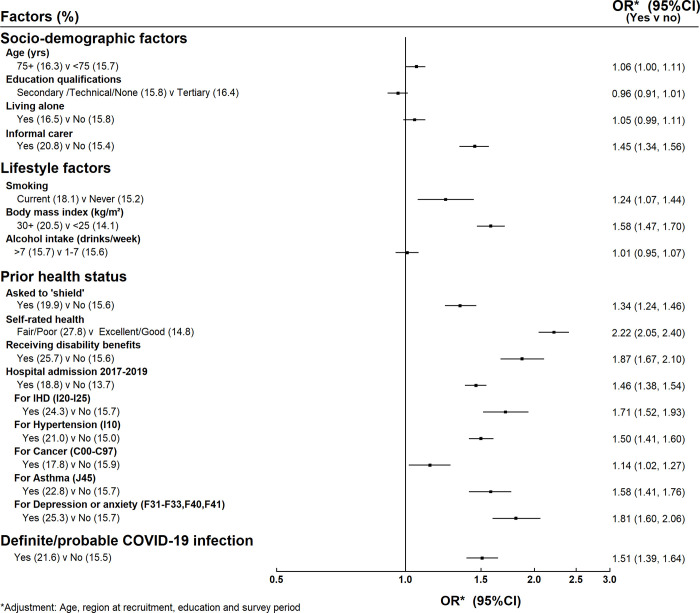
Factors associated with delaying seeking medical help.

16% of participants reported they had delayed seeking medical care for something for which they later needed treatment. The main factors related to a delay in seeking medical care were similar to those related to reporting worse physical and mental health: informal carers (21% vs 15%, adjusted OR 1.45, 95% CI 1.34–1.56), women with BMI > = 30 kg/m^2^ vs <25 kg/m^2^ (21% vs 14%, adjusted OR 1.58, 95% CI 1.47–1.70) and those in poor health prior to the outbreak (eg poor/fair self-rated health 28% vs 15%, adjusted OR 2.22, 95% CI 2.05–2.40) were all more likely to delay seeking medical care for a condition which eventually needed treatment (Fig 3).

In sensitivity analyses, where participants who reported having had a COVID-19 infection were excluded, the results remained essentially unchanged, except for a weakening of the association with smoking (S4–S6 Figs). There were no statistically significant differences in the associations between survey periods (S2–S4 Tables).

## Discussion

Among a large population-based sample of older women in the UK, surveyed during the second and third national lockdowns in 2020–21, informal carers and those with pre-existing medical conditions were most likely to report a worsening of their physical health during the COVID-19 outbreak, and this association remained after excluding those who had a COVID-19 infection. Along with women who had depression/anxiety, these groups were also most likely to report worsening mental health and to report delaying seeking medical care for a condition that eventually needed treatment.

These results are similar to the findings of other studies from the UK of an increase in mental distress during the outbreak, particularly in women, [11,13–16] those with pre-existing conditions [11,15], those living alone [11,13,16] and informal carers [17,18]. However, our results also reveal a deterioration in self-reported physical health alongside a deterioration in self-reported mental health, particularly for informal carers. A study of 5149 older adults in the English Longitudinal Study of Ageing reported that co-resident carers were more likely to report poorer self-rated health compared to those who were carers of people outside their home [19]. There is also qualitative evidence showing that carers’ well-being was impacted due to the increased burden that was placed on carers during this time, with cancellations of support and respite services [26–28], and a reduction in the use of paid home care because of the concern over introducing infection into the home [29]. Our results also show that informal carers were more likely to report delaying seeking healthcare for themselves, a finding which has also been reported by a study of US adults [30]. Overall, these findings emphasise the importance of supporting informal carers, which is recommended by NICE [31], and suggest that enhanced monitoring of informal carers’ physical health is required.

Sixteen percent of participants reported that they had delayed seeking healthcare for something that eventually needed treatment. This is in line with evidence of reductions in contacts with GPs and confirms that people were trying to self-manage symptoms in order to avoid contact with the NHS [22]. There is also evidence that after March 2021 contacts with GPs increased beyond pre-pandemic levels [32]. We found that the women who were most likely to report avoiding seeking healthcare were those in poor health, those with higher BMI and informal carers. These findings are similar to those of a UK study of 2469 middle-aged adults, the Health and Employment After Fifty (HEAF) study, where 11% of their participants reported having avoided seeking healthcare when they usually would have done so, and those who were female, overweight and in poor health were most likely to avoid seeking healthcare [24].

Strengths of this study include its large size, with 40,000 participants, and its focus on older women, who have been identified as a potentially vulnerable group. It is the second largest study after the UCL COVID-19 Social Study which collected data on 70,000 adults of any age. A further strength is that information on most factors were collected prospectively, prior to the COVID-19 pandemic, thereby reducing reporting bias. A limitation is that some of the factors, such as BMI, self-rated health and disability, were reported in 2013 and so could have changed before the pandemic, leading to some exposure misclassification. In line with most other surveys [7], there was some evidence of selection bias, given that participants who did not respond to the survey tended to be in poorer health compared with those who did respond. This may mean that the proportions reported would not reflect the proportions in the overall population. It should be acknowledged that the outcome was the participants’ perception of changes in their mental and physical health, rather than an objective measure of change in health status.

## Conclusions

About a quarter of participants reported a deterioration in their physical or mental health during the COVID-19 outbreak in the UK. The most vulnerable groups were those who were already in poor health and informal carers, as well as those who had a COVID-19 infection. This highlights the importance of supporting not only those in poor health but also their carers, in order to avoid some of the negative health consequences related to pandemic containment measures.

## Supporting information

S1 Fig Flowchart of inclusions and exclusions. (PDF)

S2 Fig Factors associated with improved physical health. (TIFF)

S3 Fig Factors associated with improved mental health. (TIFF)

S4 Fig Factors associated with worsening physical health (excluding women who have had a Covid infection). (TIFF)

S5 Fig Factors associated with worsening mental health (excluding women who have had a Covid infection). (TIFF)

S6 Fig Factors associated with delaying seeking medical help (excluding women who have had a Covid infection). (TIFF)

S1 Table Characteristics of responders and non-responders. (PDF)

S2 Table Factors associated with worsening physical health stratified by survey period (worse v stay same). (PDF)

S3 Table Factors associated with worsening mental health stratified by survey period (worse v stay same). (PDF)

S4 Table Factors associated with delaying seeking medical help stratified by survey period (yes v no). (PDF)
