# Supplementary figures and images for: Physical and mental health of 40,000 older women in England during the COVID-19 pandemic (2020–2021)
Source: PLoS One. 2024 Jul 18;19(7):e0307106. doi: 10.1371/journal.pone.0307106 (PMC11257346; doi:10.1371/journal.pone.0307106)

**S1 Fig. Flowchart of inclusions and exclusions**

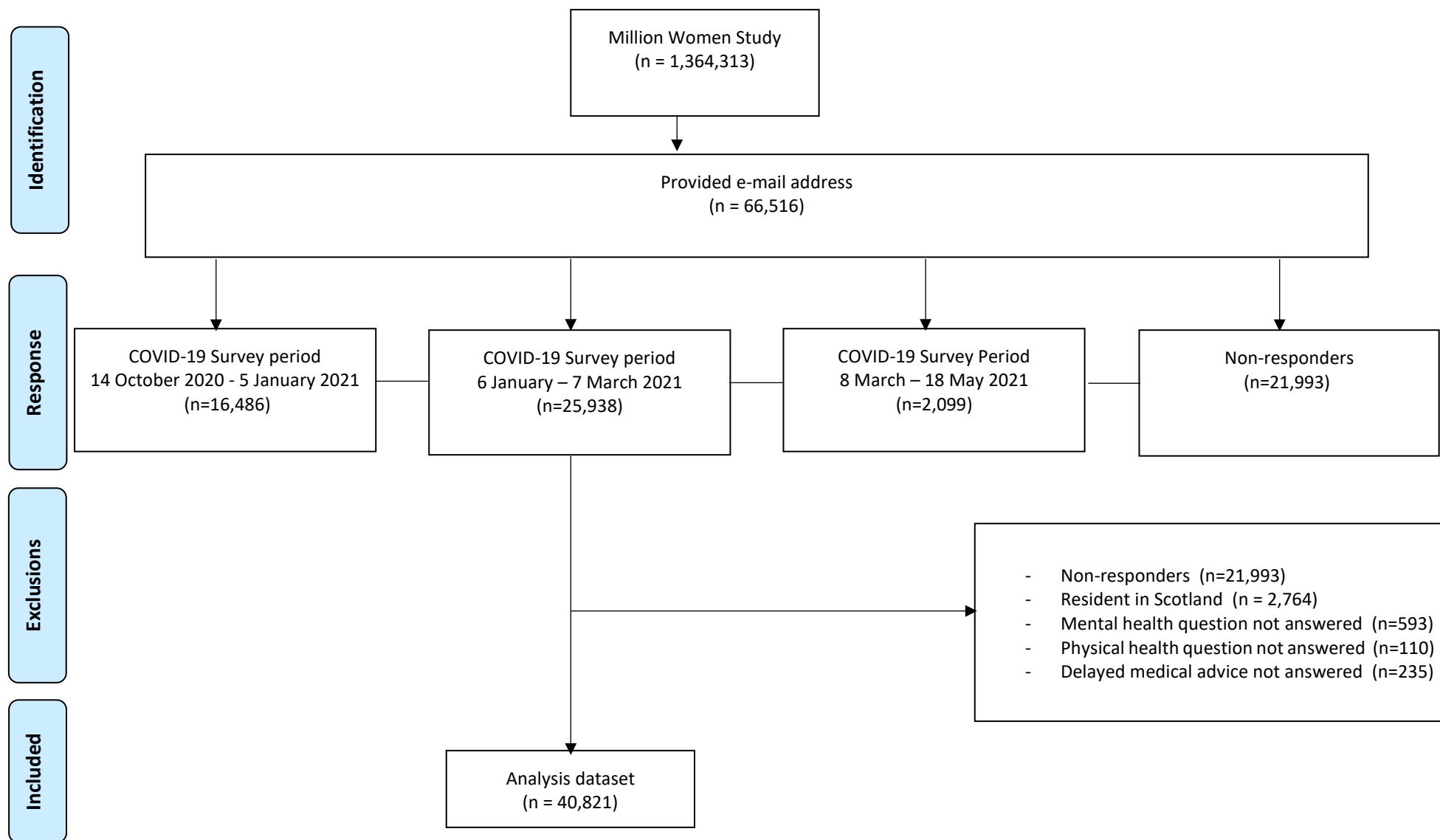

Supplement: S1 Fig — (PDF) [file pone.0307106.s001.pdf]

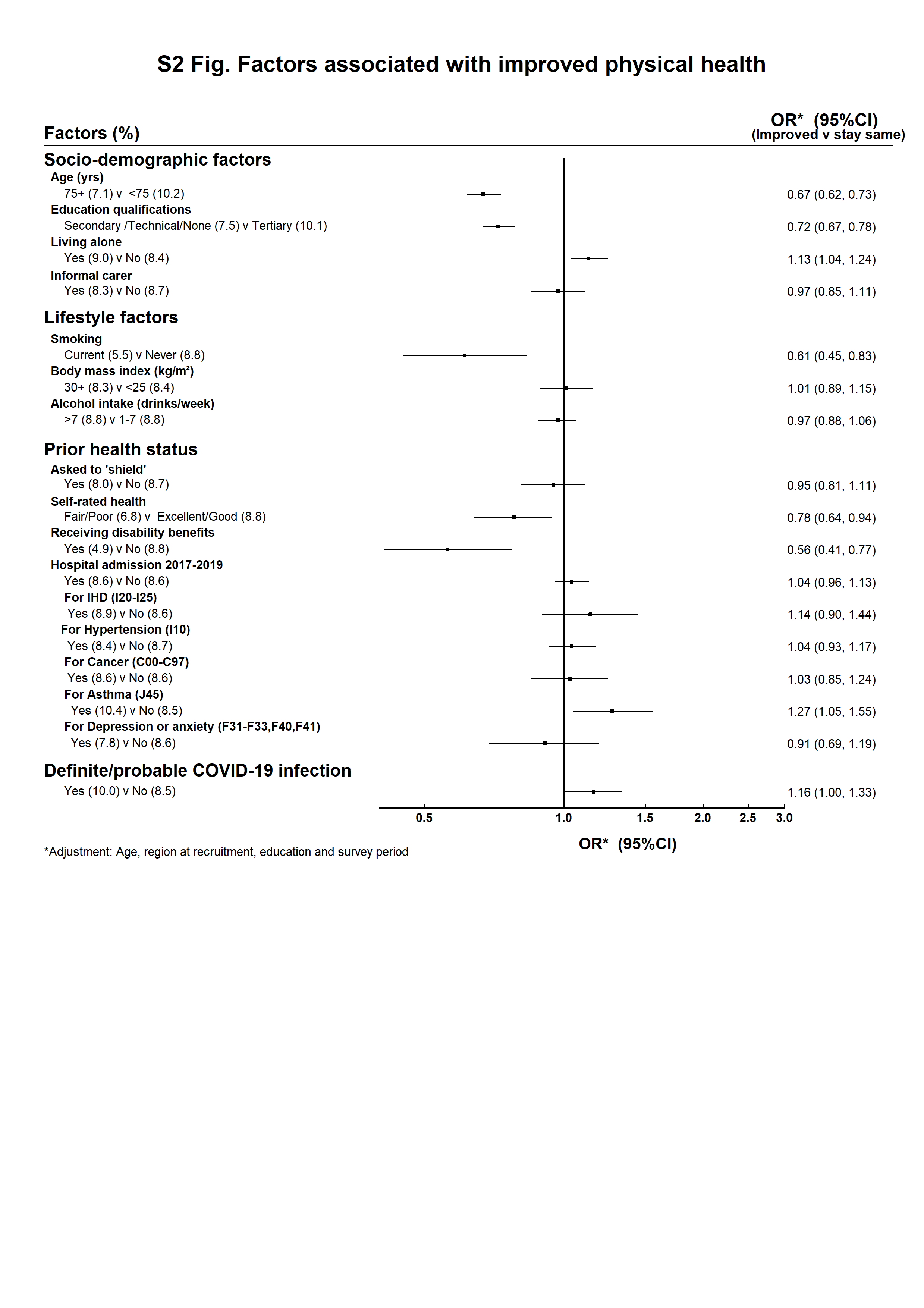

Supplement: S2 Fig — (TIFF) [file pone.0307106.s002.tiff]

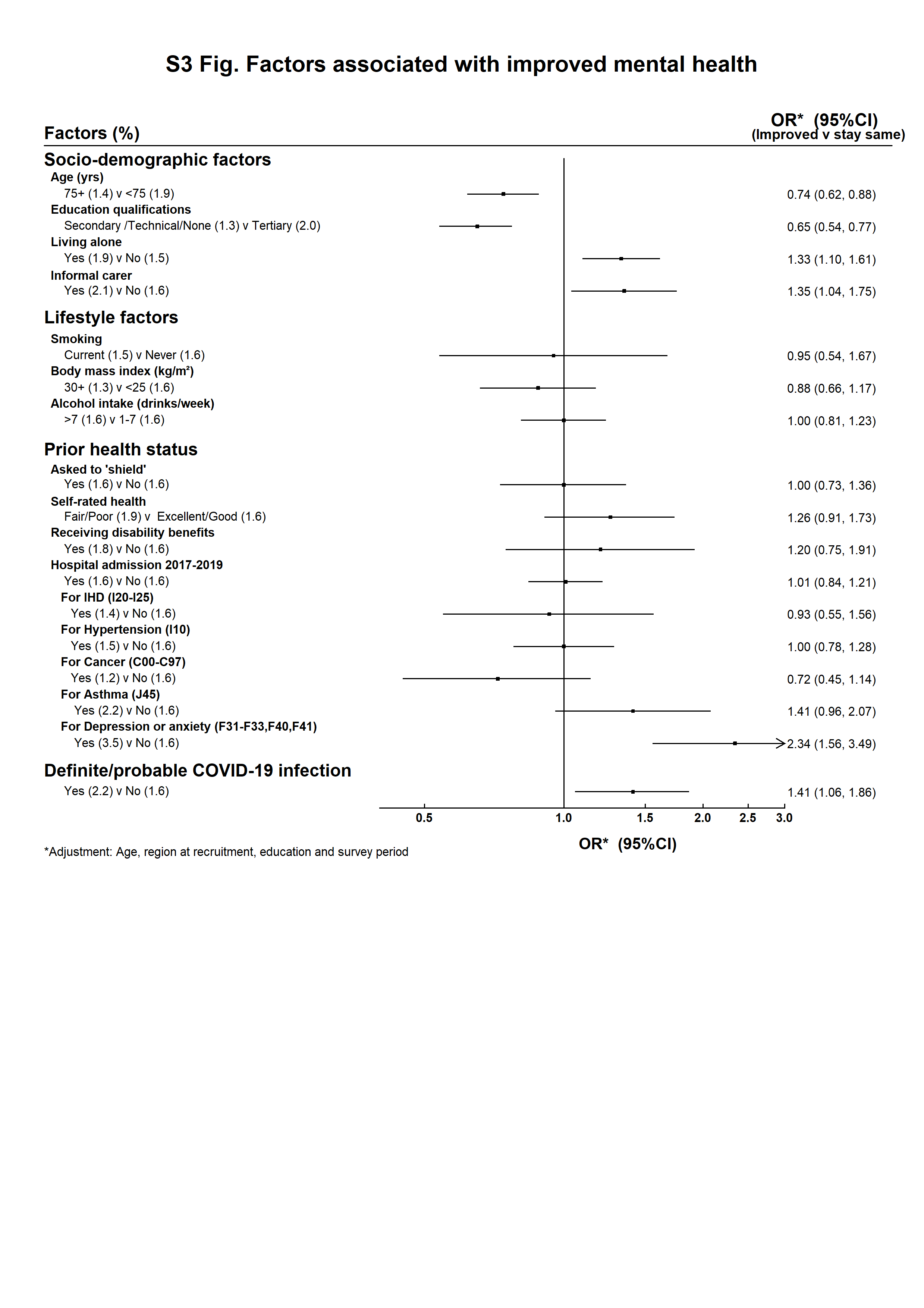

Supplement: S3 Fig — (TIFF) [file pone.0307106.s003.tiff]

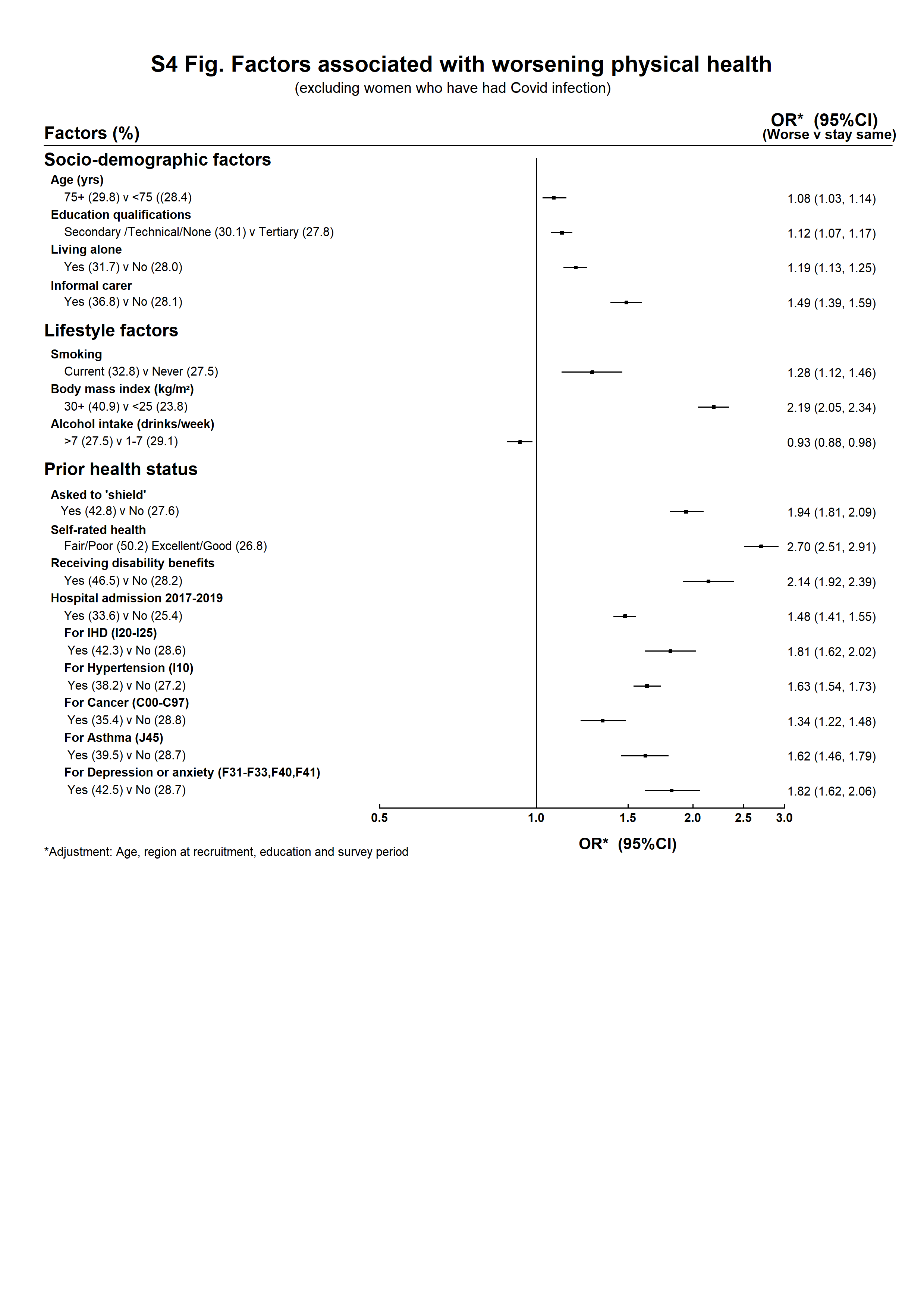

Supplement: S4 Fig — (TIFF) [file pone.0307106.s004.tiff]

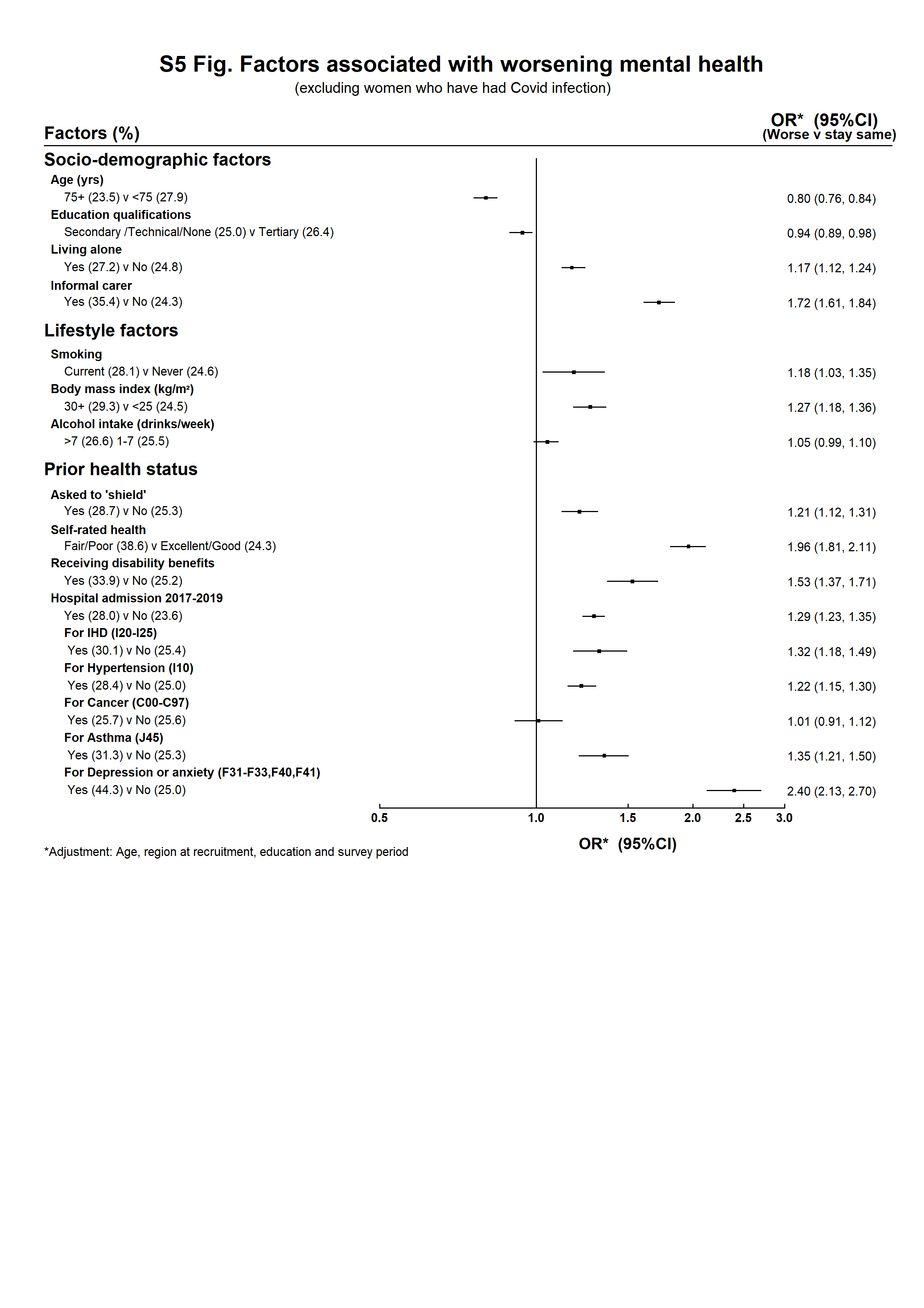

Supplement: S5 Fig — (TIFF) [file pone.0307106.s005.tiff]

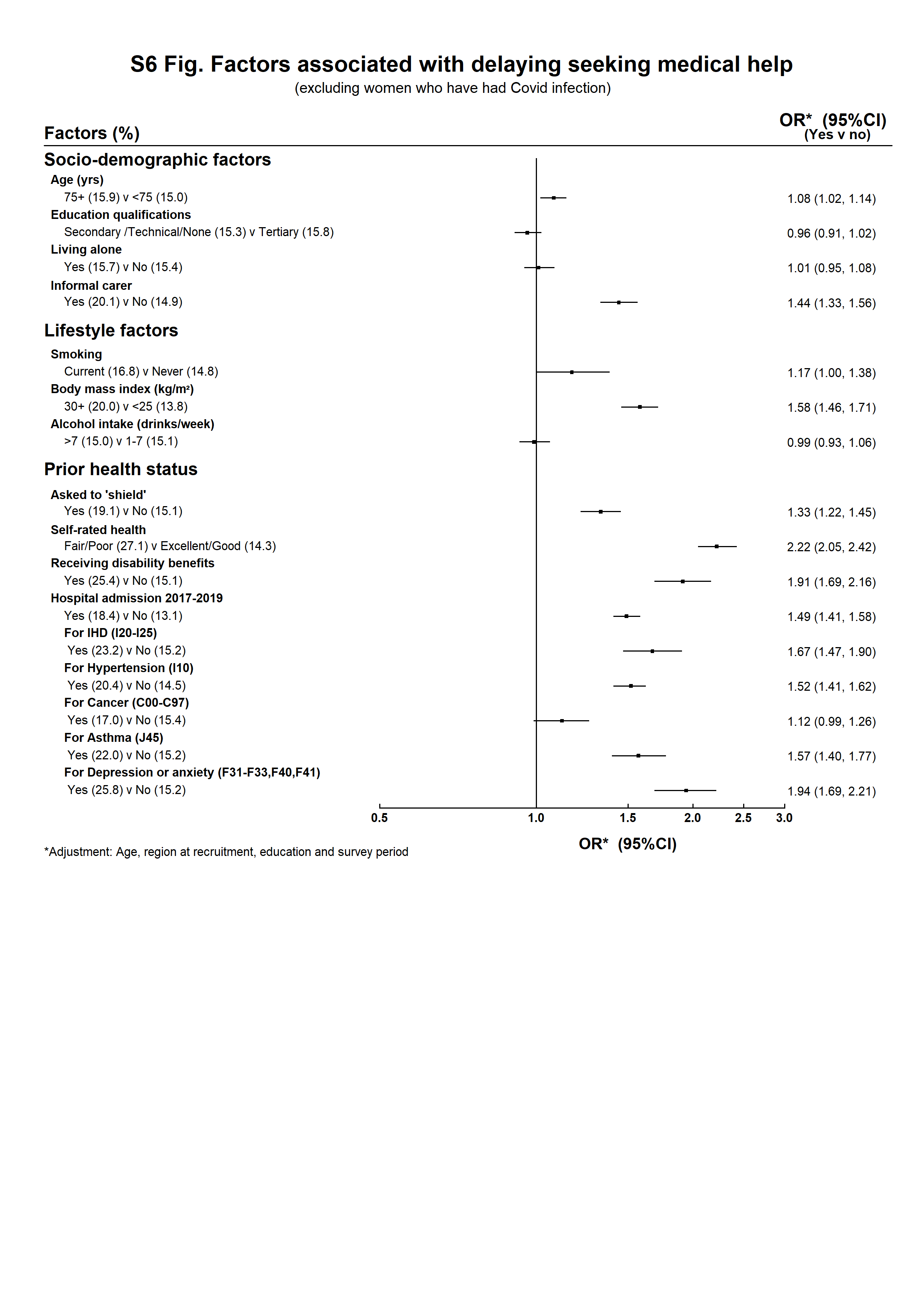

Supplement: S6 Fig — (TIFF) [file pone.0307106.s006.tiff]
