# Supplementary material for: Physical and mental health of 40,000 older women in England during the COVID-19 pandemic (2020–2021)
Source: PLoS One. 2024 Jul 18;19(7):e0307106. doi: 10.1371/journal.pone.0307106 (PMC11257346; doi:10.1371/journal.pone.0307106)
Supplement: S1 Table — (PDF) [file pone.0307106.s007.pdf]

S1 Table Characteristics of responders and non-responders

Characteristics	All responders	Non-responders	p-value*
	(n=44523)	(n=21993)	
**Socio-demographic factors**			
Age, mean (SD)	76 (4)	76 (4)	
<75, % (n)	48 (21554)	41 (9059)	< 0.001
≥75, % (n)	52 (22969)	59 (12934)	
Tertiary educational qualifications, % (n)	44 (19282)	36 (7759)	< 0.001
**Lifestyle factors**			
Current smoker in 2011-13, % (n)	3 (1341)	4 (787)	< 0.001
BMI >=30kg/m² in 2011-13, % (n)	16 (6770)	21 (4145)	< 0.001
Alcohol intake >7 drinks/week in 2011-13, % (n)	34 (14178)	32 (6095)	< 0.001
**Prior health status**			
Poor/fair self-rated health in 2011-13, % (n)	9 (3930)	15 (2944)	< 0.001
Receiving disability benefit in 2011-13, % (n)	4 (1770)	8 (1581)	< 0.001
Any hospital admission (2017-2019), % (n)	45 (19906)	50 (11073)	< 0.001
Hospital admission (2017-2019) with mention of:			
Ischaemic heart disease, % (n)	4 (1672)	6 (1230)	< 0.001
Hypertension, % (n)	17 (7410)	22 (4752)	< 0.001
Cancer, % (n)	5 (2406)	6 (1373)	< 0.001
Asthma, % (n)	4 (1971)	6 (1213)	< 0.001
Depression/anxiety, % (n)	3 (1347)	5 (1119)	< 0.001
**Additional adjustment**			
Region at recruitment in 1998			
London and South East, % (n)	30 (13560)	28 (6140)	< 0.001
South West, % (n)	23 (10144)	22 (4835)	
Midlands, % (n)	16 (7103)	16 (3554)	
North, % (n)	25 (10952)	26 (5769)	
Scotland, % (n)	6 (2764)	8 (1695)	
Survey period			
14 October 2020 - 5 January 2021	37 (16486)	24 (5260)	< 0.001
6 January - 7 March 2021	58 (25894)	61 (13472)	
8 March - 18 May 2021	5 (2143)	15 (3261)	

*derived using Pearson chi-square test
