# Supplementary material for: Physical and mental health of 40,000 older women in England during the COVID-19 pandemic (2020–2021)
Source: PLoS One. 2024 Jul 18;19(7):e0307106. doi: 10.1371/journal.pone.0307106 (PMC11257346; doi:10.1371/journal.pone.0307106)
Supplement: S2 Table — (PDF) [file pone.0307106.s008.pdf]

S2 Table Factors associated with worsening physical health stratified by survey period (worse v stay same)

Factors	SURVEY PERIOD								
	14 October 2020 - 5 January 2021			6 January - 7 March 2021			8 March - 18 May 2021		
**Socio-demographic factors**	**OR***	**95% CI**		**OR***	**95% CI**		**OR***	**95% CI**	
Age	1.09	1.01	1.17	1.06	1.00	1.12	1.07	0.87	1.30
Education qualifications	1.06	0.98	1.14	1.10	1.04	1.17	1.31	1.07	1.61
Living alone	1.27	1.17	1.37	1.14	1.07	1.21	1.36	1.10	1.68
Informal carer	1.50	1.35	1.66	1.47	1.35	1.59	1.31	0.99	1.74
**Lifestyle factors**									
Smoking									
Past v Never	1.21	1.12	1.30	1.12	1.06	1.19	1.36	1.10	1.68
Current v Never	1.32	1.07	1.63	1.31	1.11	1.54	1.87	1.11	3.15
Body mass index (kg/m^2)									
25-29 v <25	1.45	1.33	1.58	1.37	1.29	1.47	1.42	1.12	1.79
30+ v < 25	2.19	1.97	2.44	2.16	1.99	2.34	2.21	1.66	2.94
Alcohol intake (drinks/week)									
Never v 1-7	1.17	1.05	1.30	1.16	1.07	1.27	1.21	0.90	1.63
>7 v 1-7	0.92	0.84	1.00	0.94	0.88	1.00	1.09	0.86	1.38
**Prior health status**									
Asked to 'shield'	2.14	1.91	2.40	1.77	1.62	1.93	2.21	1.63	2.99
Self rated health	2.66	2.35	3.00	2.74	2.50	3.00	2.73	2.01	3.70
Receiving disability benefits	2.34	1.96	2.80	2.05	1.81	2.34	2.24	1.40	3.60
Hospital admission 2017-2019	1.49	1.39	1.61	1.46	1.38	1.54	1.40	1.15	1.70
For IHD (I20-I25)	1.92	1.61	2.28	1.72	1.50	1.98	2.25	1.38	3.68
For Hypertension (I10)	1.71	1.56	1.88	1.56	1.46	1.68	1.72	1.35	2.19
For Cancer (C00-C97)	1.20	1.02	1.41	1.42	1.26	1.59	1.35	0.90	2.02
For Asthma (J45)	1.69	1.43	1.99	1.57	1.38	1.78	1.97	1.25	3.12
For Depression/anxiety (F31-F33,F40,F41)	1.72	1.41	2.10	1.75	1.51	2.03	2.06	1.27	3.34
Definite/probable COVID infection	1.67	1.48	1.90	1.61	1.47	1.77	1.74	1.26	2.41

* Adjustment: Age, region at recruitment, education and survey period
