# Supplementary material for: Physical and mental health of 40,000 older women in England during the COVID-19 pandemic (2020–2021)
Source: PLoS One. 2024 Jul 18;19(7):e0307106. doi: 10.1371/journal.pone.0307106 (PMC11257346; doi:10.1371/journal.pone.0307106)
Supplement: S3 Table — (PDF) [file pone.0307106.s009.pdf]

**S3 Table Factors associated with worsening mental health stratified by survey period (worse v stay same)**

| | SURVEY PERIOD | | | | | | | | |
|---|---|---|---|---|---|---|---|---|---|
| **Factors** | **14 October 2020 - 5 January 2021** | | | **6 January - 7 March 2021** | | | **8 March - 18 May 2021** | | |
| **Socio-demographic factors** | **OR*** | **95% CI** | | **OR*** | **95% CI** | | **OR*** | **95% CI** | |
| Age | 0.78 | 0.72 | 0.84 | 0.80 | 0.75 | 0.85 | 0.97 | 0.79 | 1.20 |
| Education qualifications | 0.92 | 0.86 | 0.99 | 0.94 | 0.89 | 1.00 | 0.95 | 0.76 | 1.17 |
| Living alone | 1.21 | 1.11 | 1.31 | 1.18 | 1.11 | 1.26 | 1.04 | 0.83 | 1.30 |
| Informal carer | 1.76 | 1.58 | 1.95 | 1.63 | 1.50 | 1.77 | 1.87 | 1.41 | 2.48 |
| **Lifestyle factors** | | | | | | | | | |
| Smoking | | | | | | | | | |
| Past v Never | 1.13 | 1.04 | 1.22 | 1.14 | 1.08 | 1.22 | 1.08 | 0.86 | 1.35 |
| Current v Never | 1.12 | 0.90 | 1.39 | 1.19 | 1.00 | 1.40 | 2.20 | 1.30 | 3.72 |
| Body mass index (kg/m²) | | | | | | | | | |
| 25-29 v <25 | 1.05 | 0.96 | 1.14 | 1.07 | 1.00 | 1.14 | 0.94 | 0.73 | 1.20 |
| 30+ v < 25 | 1.29 | 1.15 | 1.44 | 1.26 | 1.16 | 1.37 | 1.05 | 0.78 | 1.43 |
| Alcohol intake (drinks/week) | | | | | | | | | |
| Never v 1-7 | 0.93 | 0.83 | 1.04 | 0.94 | 0.86 | 1.03 | 0.94 | 0.67 | 1.31 |
| >7 v 1-7 | 0.98 | 0.90 | 1.07 | 1.06 | 1.00 | 1.14 | 1.48 | 1.17 | 1.88 |
| **Prior health status** | | | | | | | | | |
| Asked to 'shield' | 1.21 | 1.07 | 1.37 | 1.21 | 1.10 | 1.33 | 1.33 | 0.97 | 1.84 |
| Self rated health | 1.78 | 1.57 | 2.02 | 2.05 | 1.87 | 2.24 | 2.55 | 1.88 | 3.46 |
| Receiving disability benefits | 1.70 | 1.42 | 2.05 | 1.45 | 1.27 | 1.66 | 1.59 | 0.97 | 2.59 |
| Hospital admission 2017-2019 | 1.30 | 1.21 | 1.40 | 1.27 | 1.19 | 1.34 | 1.27 | 1.03 | 1.56 |
| For IHD (I20-I25) | 1.32 | 1.10 | 1.59 | 1.34 | 1.16 | 1.55 | 1.45 | 0.87 | 2.42 |
| For Hypertension (I10) | 1.27 | 1.15 | 1.39 | 1.21 | 1.12 | 1.30 | 1.13 | 0.87 | 1.47 |
| For Cancer (C00-C97) | 0.89 | 0.74 | 1.05 | 1.11 | 0.98 | 1.25 | 1.17 | 0.76 | 1.79 |
| For Asthma (J45) | 1.41 | 1.19 | 1.67 | 1.31 | 1.15 | 1.50 | 0.99 | 0.60 | 1.65 |
| For Depression/anxiety (F31-F33,F40,F41) | 2.39 | 1.97 | 2.89 | 2.28 | 1.97 | 2.64 | 2.03 | 1.25 | 3.30 |
| Definite/probable COVID infection | 1.25 | 1.10 | 1.42 | 1.22 | 1.11 | 1.35 | 1.48 | 1.05 | 2.08 |

* Adjustment: Age, region at recruitment, education and survey period
